# In vivo Roles of Rab27 and Its Effectors in Exocytosis

**DOI:** 10.1247/csf.21043

**Published:** 2021-09-04

**Authors:** Tetsuro Izumi

**Affiliations:** 1 Laboratory of Molecular Endocrinology and Metabolism, Department of Molecular Medicine, Institute for Molecular and Cellular Regulation, Gunma University, Maebashi, Gunma 371-8512, Japan

**Keywords:** membrane trafficking, regulated exocytosis, insulin granules, pancreatic beta cells

## Abstract

The monomeric GTPase Rab27 regulates exocytosis of a broad range of vesicles in multicellular organisms. Several effectors bind GTP-bound Rab27a and/or Rab27b on secretory vesicles to execute a series of exocytic steps, such as vesicle maturation, movement along microtubules, anchoring within the peripheral F-actin network, and tethering to the plasma membrane, via interactions with specific proteins and membrane lipids in a local milieu. Although Rab27 effectors generally promote exocytosis, they can also temporarily restrict it when they are involved in the rate-limiting step. Genetic alterations in Rab27-related molecules cause discrete diseases manifesting pigment dilution and immunodeficiency, and can also affect common diseases such as diabetes and cancer in complex ways. Although the function and mechanism of action of these effectors have been explored, it is unclear how multiple effectors act in coordination within a cell to regulate the secretory process as a whole. It seems that Rab27 and various effectors constitutively reside on individual vesicles to perform consecutive exocytic steps. The present review describes the unique properties and in vivo roles of the Rab27 system, and the functional relationship among different effectors coexpressed in single cells, with pancreatic beta cells used as an example.

## Introduction

Exocytosis comprises multiple steps, from vesicle biogenesis to fusion with the plasma membrane. Rab27 and its effectors are specifically located on secretory vesicles and are thought to play versatile and critical roles in their transport from the cell interior to the vicinity of the plasma membrane (Izumi *et al.*, 2003). In fact, cells subjected to knockdown of Rab27-related molecules have demonstrated pleiotropic exocytic defects, although the underlying molecular mechanism is not well understood. Additionally, some silencing effects observed in cultured cells can be difficult to reproduce as overt phenotypic changes at whole body levels. The present review describes the unique properties of the Rab27 system and the function of each Rab27 effector with reference to the in vivo phenotypes seen in mice, humans, and their derivative cells with loss-of-function mutations. The functional relationship among different effectors expressed in the same cells is also described, with specific reference to pancreatic beta cells as an example. The findings obtained from beta cells suggest that different Rab27 effectors continuously reside on each individual vesicle and regulate distinct exocytic steps in sequence or in parallel, through specific interactions with local proteins and lipids.

### Rab27 on exocytic vesicle membrane

Among the Rab protein family members that regulate vesicular trafficking, the Rab27 subfamily members, Rab27a and Rab27b, are located on various post-Golgi vesicles in secretory cells, such as endocrine and exocrine secretory granules generated from the trans-Golgi network (TGN) and lysosome-related organelles derived from connections with endosomes (Gomi *et al.*, 2007; Tolmachova *et al.*, 2004). They also exist on vesicles providing plasma membrane for mechanical extension and surface protection and repair in non-secretory cells, for example, fusiform vesicles in the umbrella cells lining the surface of the transitional epithelium in the urinary bladder (Chen *et al.*, 2003). Mutations of the Rab27a gene cause Griscelli syndrome type 2 manifesting in partial albinism due to insufficient melanosome transfer from skin melanocytes to keratinocytes and fatal hemophagocytic disorder due to defective lytic granule exocytosis in cytotoxic T lymphocytes (Ménasche *et al.*, 2000). Rab27a-mutated *ashen* mice show similar defects in these cells (Haddad *et al.*, 2001; Hume *et al.*, 2001; Stinchcombe *et al.*, 2001; Wilson *et al.*, 2000). However, mice deficient in Rab27a, Rab27b, or both are viable under the specific-pathogen-free (SPF) breeding condition (Gomi *et al.*, 2007; Tolmachova *et al.*, 2007). No known human disease has been attributed to a Rab27b mutation. In contrast to its absolutely indispensable exocytic roles in skin melanocytes and cytotoxic T lymphocytes, Rab27 appears to play only regulatory roles in other cells by increasing the efficiency and/or fidelity of vesicle secretion. However, a broader investigation in mice deficient in Rab27a and/or Rab27b has revealed significant defects in a wide range of cells, including pancreatic beta cells (Kasai *et al.*, 2005), pancreatic acinar cells (Hou *et al.*, 2015), dendritic cells (Jancic *et al.*, 2007), platelets (Novak *et al.*, 2002; Tolmachova *et al.*, 2007), mast cells (Mizuno *et al.*, 2007), neutrophils (Johnson *et al.*, 2010), eosinophils (Kim *et al.*, 2013), osteoclasts (Shimada-Sugawara *et al.*, 2015), oocytes (Cheeseman *et al.*, 2016), and others. Further investigation will likely reveal more disorders.

Most Rabs contain a CC or CXC prenylation motif at the C terminus, and undergo geranylgeranyl isoprenoid addition, which is required for their target membrane association and proper function. This lipid modification is initiated by complex formation with Rab escort protein (REP) and is catalyzed by Rab geranylgeranyl transferase (RGGT) (Casey and Seabra, 1996). The genetic defect of REP-1 or the α-subunit of RGGT is known to cause selective underprenylation of Rab27a, leading to retinal degeneration and platelet dysfunction (Detter *et al.*, 2000; Larijani *et al.*, 2003; Seabra *et al.*, 1995). Furthermore, downregulation of REP-1, the α- or β-subunit of RGGT, or geranylgeranyl pyrophosphate synthase markedly attenuates glucose-induced insulin secretion in pancreatic beta cells (Arora *et al.*, 2012; Jiang *et al.*, 2016), as found in Rab27a-deficient *ashen* mice (Kasai *et al.*, 2005). The selective susceptibility of Rab27 may reflect its requirement for geranylgeranylation on numerous nascent vesicles after stimulus-induced fusion and the disappearance of preexisting vesicles. Despite this, the majority of Rab27 exists in a GTP-bound form and stably associates with the vesicle membrane in wild-type cells. For example, Rab27a predominantly exists in a GTP-bound form in unstimulated platelets (Kondo *et al.*, 2006). Furthermore, most Rab27a exists in membrane and detergent (1% Nonidet P-40)-insoluble particulate fractions in the pancreatic beta-cell line MIN6, although closely related Rab3a is equally present in the membrane and cytosolic fractions, but not in the particulate fraction (Yi *et al.*, 2002). Fluorescence recovery after photobleaching (FRAP) analyses indicate that Rab27a shows lower turnover and more stable localization on the vesicle membrane compared with other Rabs, including the Rab3 subfamily members (Bierings *et al.*, 2012; Handley *et al.*, 2007; Kiskin *et al.*, 2010; Robinson *et al.*, 2019). Although the precise mechanisms governing targeting of Rab27 to specific membrane compartments is unclear, Rab27 appears to be stabilized in a membrane-associated GTP-bound form once geranylgeranylated.

The guanine nucleotide binding state of Rabs is regulated by guanine nucleotide exchange factors (GEFs) and GTPase-activating proteins (GAPs). AEX-3, the *Caenorhabditis elegans* ortholog of Rab3GEP/DENN/MADD (Wada *et al.*, 1997), increases the GTP-bound Rab3 and Rab27 in heterologous cells, and its nematode mutant mislocates both Rabs in neurons (Mahoney *et al.*, 2006). Furthermore, Rab3GEP-depleted melanocytes exhibit perinuclear clustering of melanosomes and reduced levels of GTP-bound Rab27a (Figueiredo *et al.*, 2008). Thus, Rab3GEP functions as a dual GEF for Rab3 and Rab27 subfamily members in vivo. However, Rab27a can partially undergo activation and targeting in Rab3GEP-deficient melanocytes, which is suggested to occur based on the intrinsic high nucleotide exchange and low GTPase activities of Rab27a itself (Sanzà *et al.*, 2019). Rab3GEP knockout in mice is lethal due to defective neuromuscular transmission (Tanaka *et al.*, 2001), and these mice show a marked reduction in vesicular release probability without significant changes in the readily releasable pool size of their hippocampal neurons (Yamaguchi *et al.*, 2002). These phenotypes presumably reflect Rab3 dysfunction, because similar neuronal phenotypes are detected in Rab3-deficient mice (Schlüter *et al.*, 2004). On the other hand, pancreatic beta cell-specific Rab3GEP knockout mice display glucose intolerance with reduced insulin secretion (Li *et al.*, 2014), which is seen in Rab3a- and Rab27a-dificient mice (Kasai *et al.*, 2005; Yaekura *et al.*, 2003). Bialleic variations in the Rab3GEP gene in humans cause developmental delay, a broad range of endocrine and exocrine dysfunction, hematological anomalies, and neurological phenotypes, attributed to defects in vesicular trafficking and alterations in TNF-α-dependent pathways (Schneeberger *et al.*, 2020).

While GEFs for Rabs such as Rab3GEP generally contain a DENN domain (Yoshimura *et al.*, 2010), most GAPs for Rab possess a TBC domain, as found in the first GAP for Ypt/Rab GTPase, Gyp6p, in yeast (Strom *et al.*, 1993). By screening TBC domain-containing proteins, EPI64/TBC1D10A and EPI64B/TBC1D10B have been found as GAPs for Rab27 (Itoh and Fukuda, 2006). In contrast to Rab3GEP acting as a GEF toward both Rab3 and Rab27 subfamily members, EPI64 does not act as a GAP toward Rab3 members, but also acts toward Rab35 (Hsu *et al.*, 2010) and Rab8a (Hokanson and Bretscher, 2012). GTP-Rab27 appears to be maintained by high guanine nucleotide exchange activities of Rab27 itself and GEFs (for Rab27) under an excess amount of GTP relative to GDP in cells. The GDP-bound Rab27 may be subjected to rapid degradation, because Rab27a mutants mimicking either nucleotide-free or GDP-bound forms are remarkably unstable and are only minimally expressed in cells (Ramalho *et al.*, 2002; Yi *et al.*, 2002).

### The structure and function of Rab27 effectors

**Fig. 1:**
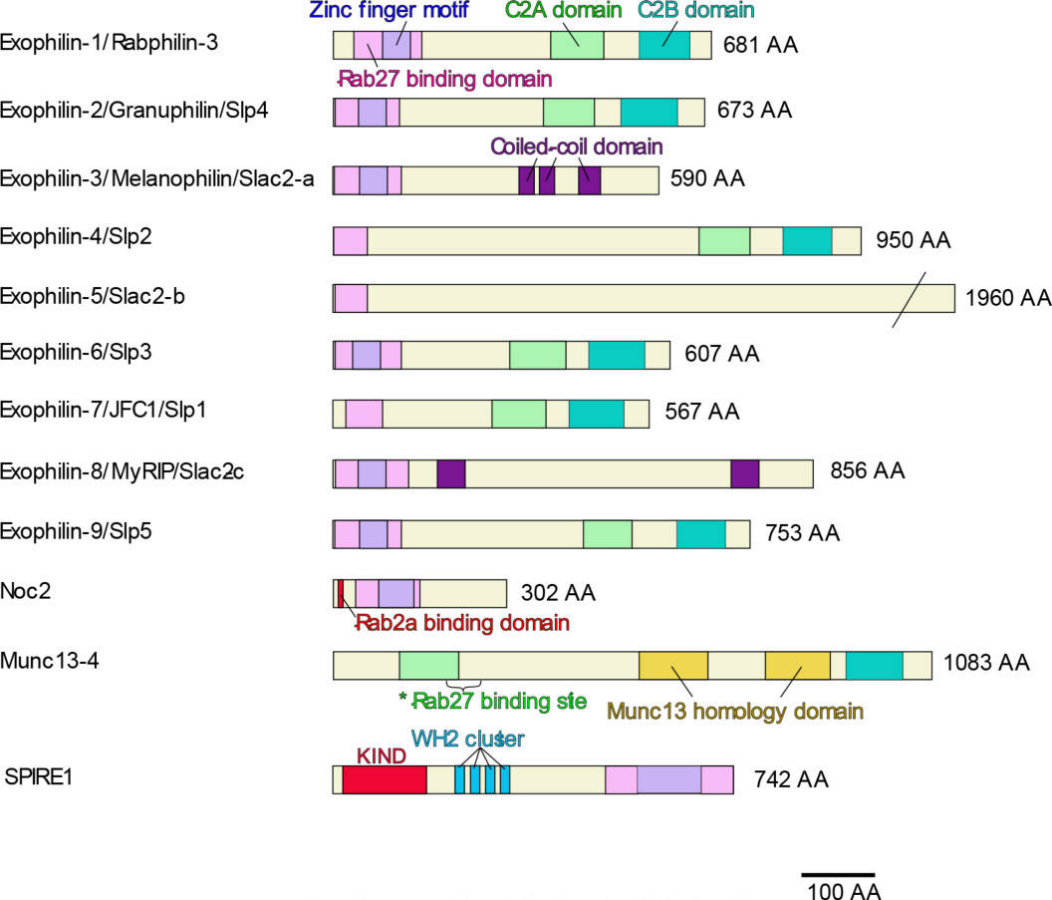
Schematic structures of Rab27 effectors. The Rab-binding domain (pink) with or without a zinc finger domain (light purple) binds Rab27, although that of rabphilin, granuphilin, or Noc2 also shows affinities to Rab3 and Rab8. Noc2 also binds Rab2a at the N-terminal region only in the presence of Rab27a. Asterisk indicates the unique Rab27 binding site of Munc13-4. The amino acid (AA) numbers on the right are those of mouse gene products.

To date, we have identified 12 effector proteins that bind the GTP-bound form of Rab27 (Fig. 1). The first Rab27 effector, granuphilin, originally discovered as a protein preferentially expressed in pancreatic beta cells relative to alpha cells, has an N-terminal Rab-binding domain and C-terminal C2 domains (Wang *et al.*, 1999). Although it has a domain structure similar to rabphilin, which was originally designated as the effector of Rab3a (Shirataki *et al.*, 1993; Yamaguchi *et al.*, 1993), considerable variations in the primary sequences of these domains between them had delayed its proper identification. Granuphilin was ultimately found to bind Rab27 by yeast two-hybrid screening and its endogenous interaction with Rab27a and/or Rab27b were confirmed in endocrine cells (Yi *et al.*, 2002; Zhao *et al.*, 2002). Although granuphilin can bind Rab3a in vitro, the interaction was scarcely detectable in these cells. On the other hand, melanophilin was discovered as a protein product of the gene whose mutation is responsible for pigment dilution in *leaden* mice (Matesic *et al.*, 2001). Melanophilin has an N-terminal sequence similar to the Rab-binding domains of granuphilin, but has no C-terminal C2 domains. Because mutations of myosin-Va or Rab27a had been known to cause similar melanosome transport defects in *dilute* and *ashen* mice and in human Griscelli syndrome type 1 and type 2 patients, respectively (Ménasche *et al.*, 2000; Mercer *et al.*, 1991; Pastural *et al.*, 1997; Wilson *et al.*, 2000), the direct interactions of melanophilin with myosin-Va and Rab27a were soon discovered to retain melanosomes within the actin networks in the cell periphery (Fukuda *et al.*, 2002a; Nagashima *et al.*, 2002; Strom *et al.*, 2002; Wu *et al.*, 2002). Although the interactions with F-actin (Kuroda *et al.*, 2003) and the microtubule plus end protein EB1 (Wu *et al.*, 2005) were also reported, they are dispensable for the peripheral melanosome distribution (Hume *et al.*, 2006, 2007). Identification of the genome by sequence similarity and of the binding protein by a yeast two-hybrid system using the Rab27-binding and C2 domains of granuphilin and melanophilin have revealed multiple candidates for Rab27 effector proteins (Fukuda and Mikoshiba, 2001; Nagashima *et al.*, 2002; Strom *et al.*, 2002), collectively termed exophilin or Slp/Slac (Fukuda, 2013; Izumi, 2007). Exophilin-7 (JFC1) and exophilin-8 (MyRIP) were also identified independently as the binding proteins for NADPH oxidase (McAdara Berkowitz *et al.*, 2001) and myosin-VIIa (El-Amraoui *et al.*, 2002), respectively. In addition, rabphilin (Mahoney *et al.*, 2006), Noc2 (Cheviet *et al.*, 2004), Munc13-4 (Neeft *et al.*, 2005; Shirakawa *et al.*, 2004), and SPIRE1/2 (Alzahofi *et al.*, 2020) have been found to function as Rab27 effectors. Although rabphilin, Noc2, and SPIRE1/2 have a conserved Rab-binding domain, Munc13-4 binds Rab27 via different primary sequences (Elstak *et al.*, 2011). The crystal structures of the Rab-binding domains have been determined for rabphilin with Rab3a (Ostermeier and Brunger, 1999), for exophilin-4 with Rab27a (Chavas *et al.*, 2008), and for melanophilin with Rab27b (Kukimoto-Niino *et al.*, 2008).

Rab27 effectors generally regulate intracellular trafficking of post-Golgi exocytic vesicles. More than half of them have two C2 domains, and likely tether or dock exocytic vesicle membranes to the target membranes by linking the two membranes via their Rab-binding and phospholipid-binding C2 domains. As stated above, however, many of them except rabphilin have considerable variations in the primary sequences, including the conserved Ca^2+^-binding residues in the precedently identified C2 domains of synaptotagmins and phospholipases (Rizo and Südhof, 1998). Rab27 effectors are also involved in organelle transport along the cytoskeleton. Melanophilin and exophilin-8, both of which lack C2 domains, have affinities to the actin-based motor proteins, myosin-Va and/or -VIIa, and function in tethering vesicles to the peripheral actin network, although the associated myosin may not necessarily act as the motor to mediate directional vesicle transport. Besides, SPIRE actin nucleator has been shown to function as a Rab27a effector to generate actin tracks for myosin-Va-dependent melanosome dispersion to the cell periphery (Alzahofi *et al.*, 2020). Although the absence of Rab27 often causes abnormal F-actin distribution in secretory cells (Ritter *et al.*, 2017; Shimada-Sugawara *et al.*, 2015; Singh *et al.*, 2013; Sinha *et al.*, 2016), it may reflect physical and/or functional interactions of Rab27 with coronins that control actin dynamics (Kimura *et al.*, 2008; Yokoyama *et al.*, 2011). The physical connections of Rab27 effectors with kinesin-1 to mediate the microtubule-based granule transport have also been reported for exophilin-6 in cytotoxic T lymphocytes and mast cells (Kurowska *et al.*, 2012; Munoz *et al.*, 2016) and for granuphilin in platelets (Adam *et al.*, 2018).

Because Rabs regulate membrane flow from one organelle to another, the concept of Rab cascades has been proposed: Rab effectors function as a GEF to activate the downstream Rab and as a GAP to inactivate the upstream Rab to generate a programmed transition from one Rab to the next (Hutagalung and Novick, 2011). However, no Rab27 effectors have been found to display intrinsic GAP or GEF activity toward other Rabs. Sequential functions of Rab27 followed by Rab3 has been suggested for acrosome exocytosis in permeabilized sperm: the Rab27a-rabphilin complex recruits another Rab3 GEF, GRAB/Rab3IL1 (Luo *et al.*, 2001), to activate Rab3a (Bustos *et al.*, 2012; Quevedo *et al.*, 2019). However, it remains to be determined whether this Rab27-Rab3 cascade really functions in intact sperm, given that GRAB displays GEF activity toward Rab8 but not to Rab3 in vitro (Yoshimura *et al.*, 2010), and acts as a Rab8 GEF and a Rab11 effector in neurons (Furusawa *et al.*, 2017).

I summarize the in vivo function of each Rab27 effector below mainly based on the phenotypic changes by knockdown or knockout rather than overexpression or expression of a dominant-negative form. Except for exophilin-6 and -9, mice deficient in the single effector have been generated but the condition is not lethal. These mice do not abrogate secretion completely in many exocytic pathways, which supports the hypothesis that each vesicle may not necessarily follow the same path before fusion (Izumi, 2011). Here, I introduce the function of Rab27 effectors not expressed in beta cells. The Rab27 effectors expressed in beta cells will be described and discussed in detail in the following chapters.

#### Rabphilin/Exophilin-1

Rabphilin, originally identified as the binding partner of Rab3a in the rat brain (Shirataki *et al.*, 1993), was later found to function as a Rab27 effector at least in neurons of *C. elegans* (Mahoney *et al.*, 2006). Although rabphilin-knockout mice were initially reported to show no overt abnormalities in synaptic transmission in contrast to Rab3a-knockout mice (Schlüter *et al.*, 1999), they did display accelerated recovery of depressed synaptic responses, which was interpreted as suggesting that rabphilin has the ability to suppress rebound hyperexcitability after bursts of activity (Deák *et al.*, 2006). Silencing of rabphilin in neurons has also been reported to reduce the surface localization of synaptic GluN2A and NMDA receptor-mediated currents at postsynaptic sites (Stanic *et al.*, 2015). In neutrophils, rabphilin is involved in integrin activation, adhesion to endothelial cells, and infiltration during inflammatory responses, although these functions are mediated via the interaction of phosphorylated rabphilin with Rab21 (Yuan *et al.*, 2017).

#### Exophilin-4/Slp2

Exophilin-4 knockdown has been reported to decrease the number of melanosomes in the cell periphery of the melanoma cell line melan-a (Kuroda and Fukuda, 2004). It also impairs lumen formation in the kidney tubular epithelial cell line MDCK (Gálvez-Santisteban *et al.*, 2012) and in human umbilical vein endothelial cells (HUVECs) (Francis *et al.*, 2021). However, the only reported phenotypic features of exophilin-4 knockout mice are modest decreases in the number of mucin granules docked to the plasma membrane and mucus secretion at an unstimulated state in gastric primary cells (Saegusa *et al.*, 2006). It is unknown whether exophilin-4 deficient animals actually manifest overt abnormalities corresponding to the findings observed in cultured cells. The Rab27a mutation that impairs both the exophilin-4 and Munc13-4 interactions but retains the melanophilin interaction causes hemophagocytic lymphohistiocytosis without albinism (Ohishi *et al.*, 2020), which suggests that, in contrast to melanophilin, exophilin-4 is dispensable for melanosome transfer to keratinocytes.

#### Exophlin-5/Slac-b

Genetic mutation of exophilin-5 causes an autosomal recessive form of epidermolysis bullosa simplex that results in skin fragility and erosions (McGrath *et al.*, 2012). Although the molecular pathogenesis is largely unknown, exophilin-5-deficient keratinocytes exhibit defects in cell adhesion and reduced secretion of extracellular vesicles containing extracellular matrix proteins (Bare *et al.*, 2020), consistent with the reported role of exophilin-5 in exosome secretion (Ostrowski *et al.*, 2010). It has recently been shown that exophlin-5 knockout mice exhibit increased allergic airway inflammation (Okunishi *et al.*, 2020). Exophilin-5 is preferentially expressed in lung epithelial cells and IL-5/IL-13-producing pathogenic Th2 cells, which express IL-33 and IL-33 receptor, respectively. Exophilin-5 deficiency increases stimulant-dependent damage and IL-33 secretion by lung epithelial cells, presumably due to epithelium fragility, and also enhances IL-5/IL-13 production from a subset of pathogenic Th2 cells in response to T-cell receptor and IL-33 stimulation. Exophlin-5 prevents the overactivation of pathogenic Th2 cells by regulating translocation of vesicles containing Nox2, a phagocyte-type NADPH oxidase generating reactive oxygen species, to the plasma membrane. This finding may account for the genetic link between Rab27a single nucleotide polymorphism and fractional enhanced nitric oxide levels, a marker of eosinophilic airway inflammation in humans (Bouzigon *et al.*, 2015).

#### Exophilin-6/Slp3

Silencing of exophilin-6 has recently been shown to accelerate neuronal migration, which causes abnormal distribution of deep-layer neurons in brain organoids and reduces presynaptic neurotransmitter release in human embryonic stem cell-derived neurons (Dong *et al.*, 2021).

#### Munc13-4

Although the Munc13 protein family generally functions in priming secretory vesicles for exocytosis, among the Munc13 isoforms only Munc13-4 can bind Rab27. Genetic defects in Rab27a and Munc13-4 lead to Griscelli syndrome type 2 and familial hemophagocytic lymphohistiocytosis type 3, respectively, both of which induce systemic inflammation due to defective lymphocyte cytotoxicity and resultant uncontrolled T-lymphocyte and macrophage activation (Feldmann *et al.*, 2003; Ménasche *et al.*, 2000). However, there is no such fatal hemophagocytic disorder in Munc13-4-deficient *Jinx* mice under the SPF condition (Crozat *et al.*, 2007), as in the case of Rab27a-deficient *ashen* mice. Munc13-4-deficiency induces exocytic defects not only in cytotoxic T lymphocytes but also in a broad range of hematopoietic cells, such as NK cells (Wood *et al.*, 2009), neutrophils (Brzezinska *et al.*, 2008), platelets (Ren *et al.*, 2010), and mast cells (Rodarte *et al.*, 2018; Singh *et al.*, 2013). In cytotoxic T lymphocytes, Munc13-4 mediates the assembly of Rab11-positive recycling endosomal vesicles and Rab27a-positive late endosomal vesicles in a Rab27-independent manner before cell activation (Ménager *et al.*, 2007). After T lymphocyte-target cell recognition that induces independent polarization of these endosomal vesicles and cytotoxic granules to the immunological synapse, the Rab27a-Munc13-4 complex appears to prime cytotoxic granules for exocytosis. Differential functions between Rab27a and Munc13-4 are also reported in neutrophils (Monfregola *et al.*, 2012). Further, Munc13-4 was subsequently shown to bind Rab11 (Johnson *et al.*, 2016).

### Roles of Rab27 effectors in pancreatic beta cells

Pancreatic beta cells express Rab27a, but not Rab27b (Kasai *et al.*, 2005; Yi *et al.*, 2002; Zhao *et al.*, 2002), and also express multiple effectors such as Noc2, exophilin-8, granuphilin, exophilin-7, and melanophilin at significant levels as described below. The following sections introduce findings in pancreatic beta cells, with reference to those reported in other cells.

#### Noc2 for granule maturation

Noc2 is a small protein without a distinct functional domain besides the Rab3/27-binding domain (Kotake *et al.*, 1997). Noc2 knockout mice exhibit impaired secretion of insulin and amylase from pancreatic endocrine and exocrine cells, respectively (Matsumoto *et al.*, 2004), although the mechanism by which Noc2 acts on the corresponding granules is unknown. It has been shown that Noc2 binds Rab2a at the region N-terminal to the Rab3/27 binding domain (Matsunaga *et al.*, 2017). Moreover, Noc2 interacts with Rab2a only in the presence of Rab27a and knockdown of Rab2a eliminates Noc2 expression even in the presence of Rab27a in beta cells. These findings suggest that both Rabs are essential for Noc2 function. In *C. elegans*, Rab2 and its effectors, such as RIC-19 (ICA69 in mammals), RUND-1, and CCCP-1, function in the sorting of cargos during granule maturation (Ailion *et al.*, 2014; Edwards *et al.*, 2009; Sumakovic *et al.*, 2009). The mutants of these molecules inappropriately sort the soluble and transmembrane cargo proteins to endosomes, but do not affect the number and distribution of granules, which indicates that Rab2 and its effectors are not involved in granule biogenesis per se but act in proper sorting of newly synthesized cargo proteins into nascent granules. Although Rab27a and Noc2 are localized on insulin granules throughout the cytoplasm of beta cells, the ternary Rab2a-Noc2-Rab27a complex is specifically localized on perinuclear proinsulin-containing immature granules (Matsunaga *et al.*, 2017). Furthermore, although knockdown of any component of the ternary complex markedly inhibits glucose-stimulated insulin secretion, only knockdown of Rab2a or Noc2, and not that of Rab27a, impairs processing of proinsulin to insulin. It seems that Noc2 plays a regulatory role in the transition between the granule cargo sorting steps regulated by Rab2a and the late exocytotic steps regulated by Rab27a, although the precise interconnecting mechanism remains unknown. Apart from these findings, it has been reported that pancreatic acinar cells from Noc2 knockout mice exhibit insufficient Ca^2+^ signals in response to physiological concentrations of secretagogues, although the underlying mechanism is unknown (Ogata *et al.*, 2012).

#### Exophilin-8/MyRIP/Slac2-c for granule capture within the actin cortex

Secretory granules budding from the TGN pass through the F-actin-rich cell cortex before reaching the plasma membrane. It has been suggested that newly formed immature granules are transported in a microtubule-dependent manner to the cell periphery and are then trapped within the actin cortex (Rudolf *et al.*, 2001), although all granules may not be processed through these cytoskeleton-based pathways. Therefore, cortical actin can act as both a mechanical barrier to the exocytic site and a reservoir to accumulate a granule pool for exocytosis in the cell periphery. Exophilin-8 appears to anchor granules within the cortical actin in various secretory cells (Desnos *et al.*, 2003; Huet *et al.*, 2012; Mizuno *et al.*, 2011; Nightingale *et al.*, 2009). In fact, it has been observed to bind myosin-VIIa by yeast two-hybrid screening (El-Amraoui *et al.*, 2002) and/or myosin-Va expressed in heterologous cells (Desnos *et al.*, 2003; Fukuda and Kuroda, 2002b), although neither interaction was demonstrated as the endogenous protein complex. The endogenous interaction between exophilin-8 and myosin-Va was later found in HUVECs (Rojo Pulido *et al.*, 2011), but this interaction does not appear to play a significant role in mediating the function of exophilin-8 in these cells (Conte *et al.*, 2016). Furthermore, there was no evidence supporting physiological interactions between these two proteins either in a melanocyte cell line harboring the melanocyte isoform of myosin-Va (Kuroda and Fukuda, 2005) or in pancreatic beta cell lines harboring the brain-type isoform of myosin-Va (Brozzi *et al.*, 2012; Waselle *et al.*, 2003). It has recently been found that exophilin-8 captures insulin granules in the actin cortex via indirect interaction with myosin-VIIa through RIM-BP2 (Fan *et al.*, 2017). Because RIM-BP2 further associates with RIM, Munc13-1, and the Ca^2+^ channel Ca_v_1.3, the exophilin-8-RIM-BP2 complex can act as a scaffold by linking a pool of granules within the F-actin network to exocytic machinery below or on the plasma membrane. Indeed, exophilin-8-null mice show glucose intolerance and lower insulin secretion. Furthermore, beta cells lacking exophilin-8 or disrupting the complex markedly decrease both peripheral accumulation and exocytosis of insulin granules. The molecular nature of myosin-VIIa identified in the complex is currently unknown, because it shows a molecular mass of ~170 kDa in gels, which is much smaller than its authentic mass of ~260 kDa. The Rab27b/exophilin-8/myosin-VIIa complex also plays a role in bacterial clearance from the bladder epithelium by collaboration with the Rab11a/FIP3/dynein complex (Miao *et al.*, 2017). Taken together, it seems that exophilin-8 physiologically functions with myosin-VIIa, but not with myosin-Va, in most cells.

#### Granuphilin/Exophlin-2/Slp4 for granule docking to the plasma membrane

Granuphilin-knockout mice exhibit enhanced glucose tolerance in vivo with increased insulin secretion in both basal and stimulated states, despite the loss of granules attached to the plasma membrane evident under electron microscopy (Gomi *et al.*, 2005). Similar phenotypic changes are found in pituitary endocrine cells (Gomi *et al.*, 2007). Although the loss of docked granules is consistent with the general roles of Rab effectors in tethering vesicle membranes to target membranes, the enhanced exocytosis in the absence of docked granules may be counterintuitive because stable predocking is generally thought to be an essential step for subsequent granule fusion. However, some confusion may arise in the use of the term ‘docking’, which has been used to refer to both the morphological tethering between the two membranes and the molecular assembly of facing v- and t-SNARE proteins (Izumi *et al.*, 2007). This is unproblematic in the case of synaptic vesicle exocytosis that proceeds within milliseconds after stimulation, since the two processes are completed before stimulation and thus may be considered together. However, in the case of much slower granule exocytosis, plasma membrane docking and fusion machinery assembly should be considered separately. In fact, granuphilin morphologically docks granules to the plasma membrane and simultaneously prevents those granules from fusing by interacting with the closed form of syntaxin-1a, -2, or -3 that cannot form a complex with other SNARE partners (Fukuda *et al.*, 2005; Torii *et al.*, 2004, 2002; Wang *et al.*, 2011). This mechanism plays an important role in restricting spontaneous fusion, because granuphilin-null beta cells exhibit increased insulin secretion in a resting state (Gomi *et al.*, 2005; Kasai *et al.*, 2008). Total internal reflection fluorescence (TIRF) microscopy visualizing fluorescently labeled granules beneath the plasma membrane reveals that granuphilin-positive granules are generally immobile in a resting state and fusion-reluctant in a stimulated state compared with granuphilin-negative granules (Mizuno *et al.*, 2016). Considering that TIRF microscopy specifically illuminates fluorescence within 100–200 nm of the coverslip and that the radius of insulin granules is 150–200 nm, the positive granules should harbor granuphilin in their hemisphere that is closer to the plasma membrane and likely form a complex with syntaxin to become immobilized on the plasma membrane. Although the exact mechanism by which this fusion-inhibitory complex is disassembled is unknown, a priming factor such as Munc13 family proteins may convert the closed form of syntaxin into the open form, as occurs in synaptic vesicle exocytosis (Richmond *et al.*, 2001).

Apart from the endocrine cells from granuphilin-knockout mice, other cells subjected to granuphilin-knockdown often suppress exocytosis of vesicles, such as exosomes in HeLa cells (Ostrowski *et al.*, 2010), secretory lysosomes in osteoblastic cells (Kariya *et al.*, 2011), vesicles forming apical membrane lumen in epithelial cells (Gálvez-Santisteban *et al.*, 2012; Vogel *et al.*, 2015), Weibel-Palade bodies (WPBs) in endothelial cells (Bierings *et al.*, 2012), dense granules in platelets (Hampson *et al.*, 2013), and lysosomes for plasma membrane repair in HeLa cells (Encarnacao *et al.*, 2016). Although none of the corresponding defective phenotypes have been documented in granuphilin-knockout mice, and some of these functions are mediated via Rab3 and Rab8 to which granuphilin has affinities, granuphilin seems to promote exocytosis simply by tethering vesicles to the plasma membrane in these constitutive and/or relatively chronic secretory processes, such as membrane biogenesis/repair and blood coagulation/homeostasis. In contrast, granuphilin strictly gates exocytosis of docked vesicles in endocrine cells where excess hormone secretion is highly consequential, such as in fatal hypoglycemia. Accordingly, the expression of granuphilin in beta cells, which affects the level of insulin secretion, is regulated multilaterally. Granuphilin is downregulated by the transcriptional repressor, ICER, which is induced by chronic hyperglycemia and cAMP-raising agents (Abderrahmani *et al.*, 2006). On the other hand, microRNA-9 expressed in beta cells increases the level of granuphilin by diminishing the expression of Onecut-2 that represses the transcriptional activity of the granuphilin promoter (Plaisance *et al.*, 2006). The granuphilin promoter is activated by SREBP-1c, a transcription factor controlling fatty acid synthesis, which may account for lipotoxicity-induced, impaired insulin secretion in diabetes (Kato *et al.*, 2006). Granuphilin is not expressed in pancreatic alpha cells that secrete the insulin-counter hormone, glucagon (Wang *et al.*, 1999). In the pancreatic alpha-cell line αTC1.6, exophilin-4, rather than granuphilin, appears to gate glucagon granule exocytosis by targeting them to the plasma membrane through the Ca^2+^-inhibitory phospholipid-binding activity of its C2A domain (Yu *et al.*, 2007). Further, granuphilin is known to be upregulated in a defined brain region specifically in male mice and controls sexually dimorphic behaviors (Xu *et al.*, 2012).

#### Exophilin-7/JFC1/Slp1 for undocked granule exocytosis

**Fig. 2:**
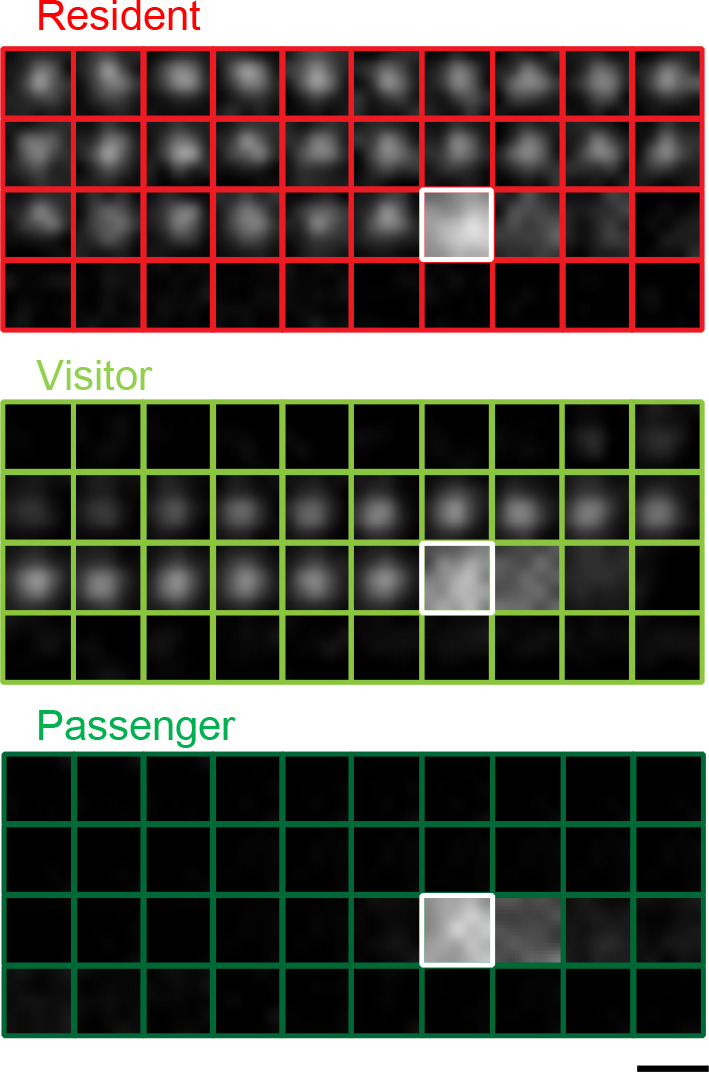
Insulin granule exocytosis in a monolayer of mouse pancreatic islet cells expressing insulin-enhanced green fluorescent protein under TIRF microscopy. Fused insulin granules imaged by TIRF microscopy are categorized into three types: residents, visitors, and passengers, depending on their distinct prefusion behaviors. Shown are examples of residents, visitors, and passengers, which morphologically represent granules that fuse with docking before stimulation, docking during stimulation, and without stable docking, to the plasma membrane, respectively. See (Wang *et al.*, 2020) for criteria of the categorization. Note that morphological docking under TIRF microscopy does not necessarily indicate the molecular tethering of granules to the plasma membrane. Sequential images were acquired every 103 milliseconds. The white boxes indicate the time of the beginning of fusion. Bar, 1 μm.

Granules recruited from the cell interior to the vicinity of the plasma membrane during or after stimulation could sustain exocytosis for a longer period. Direct observation of fluorescent insulin granule exocytosis in living pancreatic beta cells under TIRF microscopy has indeed revealed fusion not only from predocked granules but also from newly recruited granules (Kasai *et al.*, 2008; Ohara-Imaizumi *et al.*, 2004; Shibasaki *et al.*, 2007) (Fig. 2). Although exophilin-7-null mice show normal glucose tolerance and glucose-stimulated insulin secretion, their beta cells display impaired exocytosis in response to stronger secretagogues, specifically from granules that have not been predocked to the plasma membrane (Wang *et al.*, 2013). Although exophilin-7 does not appear to interact with SNARE proteins, it appears to promote tethering of granules to the plasma membrane via the affinity of its C2A domain to phospholipids. Exophilin-7 is essential for azurophilic granule exocytosis in neutrophils and may exclude polymerized actin from areas surrounding the granule via interaction with the RhoA GAP, Gem-interacting protein (Johnson *et al.*, 2012). Exophilin-7-deficiency causes increased RhoA activity and actin polymerization to prevent azurophilic granules from traversing cortical actin. If exophilin-7 similarly functions in beta cells, it may facilitate the access of undocked granule to the plasma membrane through disassembly of the cortical actin network, although the mechanism by which it directs them to the plasma membrane is unknown. Exophilin-7 has also been shown to be involved in microtubule-based anterograde transport of the neurotrophin receptor TrkB in neuronal axons via direct interaction with TrkB, the complex of which further interacts with CRMP-2 and CRMP-2-associated kinesin-1 (KIF5) (Arimura *et al.*, 2009). However, similar axonal transport of TrkA has been shown to use Rab3GEP that binds Rab3 and kinesin-3 (KIF1A) (Niwa *et al.*, 2008; Tanaka *et al.*, 2016). Although the functional differentiation between the Rab27-kinesin-1 and Rab3-kinesin-3 systems is unknown, a recent study indicates that the axonal anterograde transport of TrkB requires Rab6 and the combined activity of kinesin-1 and kinesin-3 (Zahavi *et al.*, 2021).

#### Melanophilin/Exophilin-3/Slac2-a for undocked granule exocytosis

As previously described, the role of melanophilin in skin melanocytes has been firmly established by both genetic and biochemical findings. Its functional loss leads to clustering of melanosomes near the perikaryotic region, which prevents their transfer to neighboring keratinocytes and causes hypopigmentation in both *leaden* mice and patients with Griscelli syndrome type 3 (Matesic *et al.*, 2001; Ménasche *et al.*, 2003). In fusiform vesicles of urothelial umbrella cells, melanophilin-deficiency reduces the integral membrane proteins, uroplakins, that play important roles in forming the permeability barrier and in the expansion/stabilization of the apical membrane (Wankel *et al.*, 2016). In beta cells, melanophilin and myosin-Va appear to lack the ability to accumulate insulin granules at the actin-rich cell periphery, in contrast to exophilin-8 and myosin-VIIa (Fan *et al.*, 2017; Wang *et al.*, 2020). However, melanophilin-mutated *leaden* mice show glucose intolerance and impaired glucose-stimulated insulin secretion (Wang *et al.*, 2020). Melanophilin promotes exocytosis of granules that are recruited from the cell interior and immediately fuse without stable docking to the plasma membrane (the passenger type in Fig. 2). Because melanophilin-deficiency reduces undocked granule exocytosis by half, the remainder could be mediated by exophilin-7 or some other mechanism. In contrast to granuphilin that interacts with the fusion-incompetent, closed form of syntaxin-1a, melanophilin selectively interacts with the fusion-competent, open form of syntaxin-4 that can assemble with other SNARE proteins. This binding characteristic could enable melanophilin to accelerate granule exocytosis without pausing at the plasma membrane. Consistent with its role in undocked granule exocytosis, melanophilin interacts with syntaxin-4 on the plasma membrane after stimulation in a Ca^2+^-dependent manner. In contrast, the interaction with myosin-Va that is also essential for melanophilin’s promotion of granule exocytosis is transiently disrupted upon stimulation in a Ca^2+^-dependent manner, which might release granules from the peripheral F-actin network. However, it is unlikely that myosin-Va functions as an active motor for granule movement to the plasma membrane, since the actin tracks are not usually organized to support persistent movement in any one direction (Hammer and Sellers, 2011) and the speed at which granules cross the evanescent field (100–200 nm) exceeds that displayed by myosin motors on actin filaments. Faster kinesin motors may be responsible for granule recruitment to the plasma membrane. In any case, those undocked granules should be located just above the evanescent field, still close to the plasma membrane, before they fuse in response to external stimulation. Melanophilin might be involved in track selection between the microtubule- and actin-based cargo transport systems, since observations of in vitro binding suggest that dephosphorylated melanophilin prefers binding to microtubules even in the presence of actin, whereas melanophilin phosphorylated by protein kinase A associates with actin (Oberhofer *et al.*, 2017).

### Functional relationship among different Rab27 effectors in the same cells

**Fig. 3:**
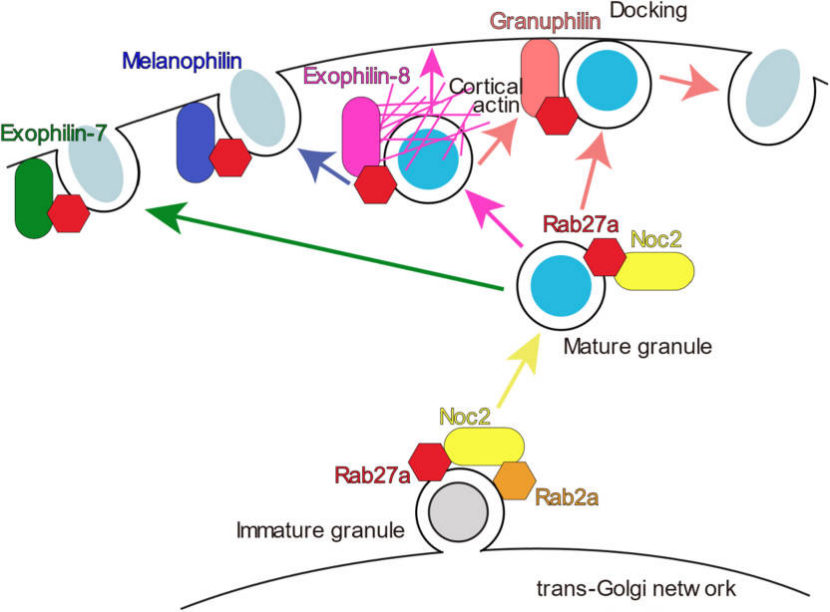
Distinct insulin granule exocytic steps regulated by different Rab27 effectors in pancreatic beta cells. Noc2 is postulated to regulate granule maturation. Exophilin-8 anchors granules in the peripheral actin network and may mediate subsequent granule exocytosis with or without granule docking to the plasma membrane. Exophilin-7 and melanophilin mediate undocked granule exocytosis, probably by different routes and mechanisms. Granuphilin mediates docked granule exocytosis. Arrows indicate some possible serial orders in which these effectors act on individual granules, although this has yet to be confirmed empirically.

As described above, each Rab27 effector regulates a distinct exocytic step in pancreatic beta cells (Fig. 3). Nonetheless, the activity is not necessarily limited to specific granules. For example, although granuphilin and Noc2 are thought to function beneath the plasma membrane and in the perinuclear region, respectively, they are both localized on insulin granules throughout the cytoplasm (Matsunaga *et al.*, 2017; Yi *et al.*, 2002). Furthermore, although granuphilin and melanophilin should separately act on different granules mediating exocytosis with or without predocking to the plasma membrane, both are colocalized on insulin granules below the plasma membrane (Wang *et al.*, 2020). GTP-binding proteins are generally subjected to GTP hydrolysis after they function to terminate their roles. In fact, marked GTP hydrolysis of Rab27 occurs after granule fusion with the plasma membrane in platelets, although this is not required for inducing granule secretion (Kondo *et al.*, 2006). This finding, however, suggests that, prior to the completion of the final fusion step leading to vesicle disappearance, the completion of an intermediate exocytic step by any Rab27 effector induces neither significant GTP hydrolysis of Rab27 nor dissociation of the involved effector from the vesicle membrane. Furthermore, in case only a subset of vesicles fuse by physiological stimulation (less than 5% of insulin granules in beta cells), the majority of effectors may remain on vesicles even after stimulation. FRAP analyses in the pheochromocytoma cell line, PC12, consistently indicate that Rab27a, granuphilin, and Noc2 show little or no exchange between secretory granules and cytosols, in contrast to Rab3a and rabphilin (Handley and Burgoyne, 2008; Handley *et al.*, 2007). These findings suggest that guanine nucleotide cycles of Rab27 by GAP and GEF, exchanges of Rab27 effectors, or even Rab cascades converting Rab27 to another Rab do not occur on the vesicle membrane in intermediate exocytic processes. In the case that multiple effectors sequentially and/or simultaneously function in the same cell, the continuous presence of GTP-bound Rab27 and multiple effectors may smooth the transition between consecutive preparatory exocytic steps. Thus, the fate of each individual vesicle may not be defined by the presence of the particular Rab27 effector per se but may be stochastically determined by its interactions with specific proteins and membrane lipids around the vesicles.

The nonspecific and stable localization on vesicles makes it difficult to judge when and where each effector functions based on the intracellular localization and membrane association status. Almost nothing is known about the coordination of the different effectors in the regulation of the distinct steps of the exocytic process. To determine whether they function in sequence or in parallel, it may be useful to analyze mice or cells simultaneously deficient in more than one effector. Thus far, only one study has investigated mice that are deficient in two effectors (Wang *et al.*, 2013). As described above, exophilin-7-knockout beta cells exhibit modestly reduced exocytosis from undocked granules, whereas granuphilin-knockout beta cells lose stably docked granules and show enhanced exocytosis from undocked granules. Because the double deficiency manifests a clearer defect in undocked granule exocytosis by exophilin-7 deficiency, it seems that exophilin-7 is involved in undocked granule exocytosis independent of granuphilin, and thus the two effectors act in parallel for undocked and docked granule exocytosis. By contrast, exophilin-8 and melanophilin appear to function in sequence, considering their roles for granules within the peripheral actin network. Namely, exophilin-8 first captures granules within a relatively broad area of the actin cortex, and then melanophilin mediates their exocytosis via interaction with syntaxin-4 on the plasma membrane. However, the precise mechanism linking these two processes is unknown. Although the cortical actomyosin II network may drive granules from the cell interior towards the plasma membrane within the actin cortex during the stimulus-induced Ca^2+^ influx (Papadopulos *et al.*, 2015), the components of the protein complexes involving the two effectors are completely different, including the associated myosin motors. Although granules anchored by exophilin-8 within the actin network can subsequently be led to melanophilin-mediated exocytosis, they may also undergo exocytosis directly via the interaction with RIM-BP2, which is linked to the exocytic machinery. Alternatively, they are subjected to granuphilin-mediated docking to the plasma membrane after release from the actin network by stimulus-induced F-actin dissolution (Fig. 3). Their actual fate can be experimentally elucidated by analyses of cells deficient in exophilin-8 and either melanophilin or granuphilin.

In regulated exocytosis, there must be at least one gating system that limits spontaneous vesicle fusion. As an extreme example, synaptic vesicles are predocked to the plasma membrane and preassemble the SNARE complex in advance in order to execute immediate exocytosis within milliseconds after stimulation. They are thought to commence fusion solely through stimulus-induced Ca^2+^ binding to the C2 domain of synaptotagmin, which releases the constraints imposed by the prefinal fusion complex consisting of SNAREs, synaptotagmin, and complexin (Brunger *et al.*, 2018; Rizo, 2018). However, the release of secretory granules that proceeds over several seconds and minutes after stimulation can be limited at much earlier, and potentially multiple, exocytic steps. In fact, two-photon fluorescence lifetime imaging analysis demonstrates that although trans-SNARE complexes accumulate in the active zone of resting presynaptic boutons, SNAREs are unassembled in resting pancreatic beta cells and are assembled only after stimulation shortly prior to insulin exocytosis (Takahashi *et al.*, 2015). Therefore, granules that appear to locate close to or to be attached to the plasma membrane in a resting state under TIRF or electron microscopy are not necessarily engaged in SNARE assembly. Rab27 effectors limit granule exocytosis differentially among secretory cells. In HUVECs, exophilin-8 restricts exocytosis of WPBs (Nightingale *et al.*, 2009), whereas granuphilin enhances it (Bierings *et al.*, 2012). Conversely, granuphilin restricts exocytosis of insulin granules, whereas exophilin-8 enhances it in beta cells, as described above. Despite the fact that exophilin-8 and granuphilin similarly promote specific exocytic processes in both cell locations, namely granule anchoring in the actin cortex and granule tethering to the plasma membrane, respectively, the opposite net effects on cargo secretion likely reflect differences in the major rate-limiting step between the two exocytic pathways. Furthermore, the limiting step can differ even within a cell: for example, granules await stimulation either by pausing beneath the plasma membrane (the resident type in Fig. 2) or by staying within the actin cortex (the passenger type in Fig. 2) in resting beta cells.

### Rab27 and its effectors in exosome release

I finally review the recent topic about the possible involvement of Rab27 and its effectors in exosome release and cancer progression. Exosomes ranging from 30 to 100 nm in diameter are generated by inwardly budding vesicles from the limiting membrane of multivesicular endosomes (MVEs) and are released upon fusion of MVEs to the plasma membrane (Colombo *et al.*, 2014; Pegtel and Gould, 2019). Exosomes have the same topology as the cell and contain selected cytoplasmic proteins, lipids, nucleic acids, and glycoconjugates, as well as membrane components. Once released, they can act on other cells and participate in diverse biological pathways, such as those for development, immunity, tissue homeostasis, cancer, and neurodegenerative diseases. Rab27 has been shown to control the exosome secretion pathway, and knockdown of either Rab27a or Rab27b inhibits this pathway in the tumor cell line HeLa B6H4 (Ostrowski *et al.*, 2010). It has been proposed that Rab27b and exophilin-5 mediate the transfer of MVEs from microtubules to the actin-rich cortex and their retention at the cell periphery, whereas Rab27a and granuphilin regulate docking leading to fusion of MVEs to the plasma membrane, although neither differential intracellular distribution nor specific complex formation of the endogenous proteins has been documented. It has also been shown that Rab27a and all exophilin-4, -5, and -6 are required for human immunodeficiency virus (HIV) production involving the exosome release in the human leukemic T-cell line Jurkat (Gerber *et al.*, 2015). In this cell line, silencing of Rab27a reduces the plasma membrane phosphatidylinositol 4.5-bisphosphate levels and impairs the recruitment of MVEs to the site of HIV assembly on the plasma membrane. Rab27a and cortactin coordinately promote exosome secretion by stabilizing the cortical actin-rich MVE docking site in the squamous cell carcinoma SCC61, although the Rab27 effector involved in this process is unknown (Sinha *et al.*, 2016). There is no consensus regarding the means, location, or specific effectors by which Rab27 regulates exosome secretion, and the mechanism may vary in different cells given the highly differential expression of Rab27 effectors. Munc13-4 also plays a pivotal role in Ca^2+^-stimulated exosome release from cancer cells, although it uses a Rab11-dependent pathway to generate MVEs competent for exosome release (Messenger *et al.*, 2018). Further, Rab7, Rab11, and Rab35 have been shown to play roles in exosome biogenesis and/or secretion (Baietti *et al.*, 2012; Hsu *et al.*, 2010; Savina *et al.*, 2002), although these Rabs may control distinct types of small extracellular vesicles derived from different subtypes of late endosomes (Colombo *et al.*, 2014).

The involvement of Rab27 in exosome biogenesis and release suggests a role in cancer progression (Li *et al.*, 2018). In fact, knockdown of Rab27a suppresses both in vitro cell growth of melanoma cells with Rab27a gene amplification (Akavia *et al.*, 2010) and in vivo tumor growth of melanoma cells where Rab27a-dependent exosome secretion educates bone marrow-derived cells to support tumor growth and metastasis (Peinado *et al.*, 2012). It has also been shown that Rab27a/b-induced exosome secretion reduces the intracellular level of tumor-suppressive microRNA and thereby increases invasion of metastatic cells (Ostenfeld *et al.*, 2014). A recent meta-analysis found that elevated expression of Rab27a and/or Rab27b is associated with poor prognosis and cancer metastasis (Koh *et al.*, 2020). However, Rab27a and Rab27b have sometimes been reported to act as tumor suppressors, because they are downregulated in colorectal and prostate cancers and their lower levels of expression are correlated with worse outcomes (Dong *et al.*, 2015; Worst *et al.*, 2017). These discrepant findings may reflect differences in the cargo molecules of exosomes released from different cancer cells.

## Perspective

Our understanding of the in vivo functions and mechanisms of action of the Rab27 effectors, with the exception of exophilin-9, has grown markedly in recent years. Nevertheless, the precise mechanisms by which individual effectors act through their molecular interactions remains elusive. Moreover, almost nothing is known about the functional hierarchy and redundancy among the different Rab27 effectors expressed in the same cell and which regulate the whole process of vesicle exocytosis. Further investigation is required to resolve these issues.
